# PRAK Interacts with DJ-1 and Prevents Oxidative Stress-Induced Cell Death

**DOI:** 10.1155/2014/735618

**Published:** 2014-10-14

**Authors:** Jing Tang, Jinghua Liu, Xue Li, Yuyun Zhong, Tianyu Zhong, Yawei Liu, Jiang Huai Wang, Yong Jiang

**Affiliations:** ^1^State Key Laboratory of Organ Failure Research, Key Laboratory of Transcriptomics and Proteomics, Ministry of Education of China, Key Laboratory of Proteomics of Guangdong Province, Southern Medical University, Guangzhou 510515, China; ^2^Nanfang Hospital, Southern Medical University, Guangzhou 510515, China; ^3^Department of Surgery, Cork University Hospital, University College Cork, Cork, Ireland

## Abstract

As a core member of p38 MAPK signal transduction pathway, p38 regulated/activated kinase (PRAK) is activated by cellular stresses. However, the function of PRAK and its downstream interacting partner remain undefined. Using a yeast two-hybrid system, we identified DJ-1 as a potential PRAK interacting protein. We further verified that DJ-1 bound to PRAK* in vitro* and* in vivo* and colocalized with PRAK in the nuclei of NIH3T3 cells. Furthermore, following H_2_O_2_ stimulation the majority of endogenous DJ-1 in PRAK^+/+^ cells still remained in the nucleus, whereas most DJ-1 in PRAK^−/−^ cells translocated from the nucleus into the cytoplasm, indicating that PRAK is essential for DJ-1 to localize in the nucleus. In addition, PRAK-associated phosphorylation of DJ-1 was observed* in vitro* and* in vivo* of H_2_O_2_-challenged PRAK^+/+^ cells. Cytoplasmic translocation of DJ-1 in H_2_O_2_-treated PRAK^−/−^ cells lost its ability to sequester Daxx, a death protein, in the nucleus, and as a result, Daxx gained access to the cytoplasm and triggered cell death. These data highlight that DJ-1 is the downstream interacting target for PRAK, and in response to oxidative stress PRAK may exert a cytoprotective effect by facilitating DJ-1 to sequester Daxx in the nucleus, thus preventing cell death.

## 1. Introduction

p38 mitogen-activated protein kinase (MAPK), a stress-activated Ser/Thr protein kinase, belongs to the MAP kinase superfamily. Study shows that p38 MAPKs are involved in cell growth [1], cell apoptosis [2], and cell cycle [3]. By regulating inflammatory processes [4], stress responses [5], transcriptional activity [6], and cytoskeletal reorganization [7], p38 MAPK plays important roles in pathological conditions including cardiomyocyte hypertrophy [8], ischemia/reperfusion injury [9], neuronal pathology [10], infectious diseases [11], wound healing, and tissue remodeling [12].

p38 regulated/activated kinase (PRAK) or MAPK activated protein kinase 5 (MK5), ubiquitously expressed in almost all human tissues, is a 471 amino acid protein with 20–30% sequence homology to the known MAPK-regulated protein kinases RSK1/2/3, MNK1/2, and MK2/3 [13]. PRAK was originally identified as a p38 MAKP-activated protein [13], but afterward work found that it was also activated by extracellular signal-regulated kinase 3/4 (ERK3/4), indicating involvement of PRAK in both p38- and ERK3/4-mediated signal transduction pathways. The evidence has suggested that PRAK/MK5 may regulate actin polymerization and cell motility and function as a tumor suppressor [14–22]. Recently, PRAK has been showed to phosphorylate several substrates including FoxO1, FoxO3, and Rheb, indicating that the biological role of PRAK is far from completely understood [23–25].

Endogenous PRAK is primarily located in the cytoplasm, whereas exogenous PRAK predominates in the nucleus [26]. A sequence analysis of PRAK revealed that PRAK contains a putative nuclear localization sequence (NLS) and a nuclear export sequence (NES), and both of them are required for the shuttling of PRAK between nucleus and cytoplasm. Following stimulation with arsenite, the nuclear PRAK was markedly reduced due to a decrease in the nuclear import of PRAK and an increase in the nuclear export of PRAK [26]. Furthermore, the nuclear import of PRAK was independent of p38 activation, whereas the nuclear export required p38-mediated phosphorylation of PRAK. However, the function of PRAK shuttling between nucleus and cytoplasm in response to different cellular stresses remains unclear.

Here, we report that DJ-1, originally found as a mitogen-dependent oncogene product [27], is a downstream interacting protein for PRAK. DJ-1 bound to PRAK both* in vitro* and* in vivo* and colocalized with PRAK in the nuclei of NIH3T3 cells. Functional studies revealed that PRAK can activate DJ-1 and help DJ-1 to localize in the nucleus. Phosphorylation of DJ-1 following H_2_O_2_ treatment was observed in PRAK^+/+^ cells but not in PRAK^−/−^ cells. Consistently, endogenous DJ-1 in PRAK^+/+^ cells was mainly located in the nucleus even after the cells were challenged with H_2_O_2_, whereas most DJ-1 in PRAK^−/−^ cells translocated from the nucleus into the cytoplasm in response to oxidative stress. As a result, DJ-1 was unable to sequester its interacting partner, a death protein Daxx in the nuclei of PRAK^−/−^ cells, thereby causing an increased cell death.

## 2. Materials and Methods

### 2.1. Plasmids and Reagents

A full-length human DJ-1 cDNA was amplified by PCR from a human adult brain cDNA library (Invitrogen) using primers 5′-GTGGATCCGCTTCCAAAAGAGCTCTGGTCATC-3′ and 5′-TGGAATTCCTAGTCTTTAAGAACAAGTGGAGC-3′ (containing restriction enzyme cleavage sites* Bam*HI and* Eco*RI) and cloned into pGEX-KG (Pharmacia) and pcDNA3-Flag (Clontech) to produce GST-tagged and Flag-tagged DJ-1. In addition, this DJ-1 cDNA was cloned into pEGFP-C2 (Clontech) and pGADT7 (Clontech) to produce GFP-tagged and AD-tagged DJ-1 using primers 5′-GTGAATTCATGGCTTCCAAAAGAGCTCTGGTCATC-3′ and 5′-TGGGATCCCGGTCTTTAAGAACAAGTGGAGC-3′ (containing restriction enzyme cleavage sites* Eco*RI and* Bam*HI). DJ-1 cDNA was cloned into pECFP-C1 (Clontech) to produce CFP-tagged DJ-1 using primers 5′-GTGAATTCTATGGCTTCCAAAAGAGCTCTGGTCATC-3′ and 5′-TGGGATCCCGGTCTTTAAGAACAAGTGGAGC-3′ (containing restriction enzyme cleavage sites* Eco*RI and* Bam*HI). A full-length human PRAK cDNA was obtained from pcDNA3-HA-PRAK (kindly provided by Dr. Jiahuai Han, The Scripps Research Institute, La Jolla) by* Nde*I and* Bam*HI dual-enzyme digestion and subcloned into pGBKT7 and pET-14b to produce DBD-PRAK and His-tagged PRAK fusion protein. This PRAK cDNA was also cloned into pEYFP-C1 (Clontech) to produce YFP-tagged PRAK fusion protein using primers 5′-TAGAATTCATCGGAGGAGAGCGACATGGACA-3′ and 5′-TAGGATCCTTATTGGGATTCGTGGGACGT-3′ (containing restriction enzyme cleavage sites* Eco*RI and* Bam*HI). All DNA plasmids were isolated and purified with Qiagen Endo-free Plasmid Maxi Kit. All other chemicals, unless indicated, were from Sigma-Aldrich.

### 2.2. Yeast Two-Hybrid Screening

The Matchmaker GAL4 Two-hybrid System 3 (Clontech, protocol PT3247-1) was used to screen for proteins that interact with PRAK. pGBKT7-PRAK was transformed into the yeast strain AH109 as the bait, and a human heart cDNA library previously transformed into the yeast strain Y187 (Clontech) was used as the prey. Approximately 1 × 10^6^ transformants were screened. After being mated, the mixtures of the bait and the prey were plated onto SD/-Ade/-His/-Leu/-Trp/X-*α*-gal plates and allowed to grow at 30°C for 4–6 days. The yeast colonies were assayed for *β*-galactosidase activity using a colony-lift filter and positive clones were subjected to sequencing.

### 2.3. Yeast Two-Hybrid Interaction Assay

Plasmids pGBKT7-PRAK and pGADT7-DJ-1 were transformed into the yeast strains Y187 and AH109, respectively. Yeast clones of AH109 expressing AD-DJ-1 and Y187 expressing DBD-PRAK were mixed and mated at 30°C for 24 hrs. To select the diploid, mated mixtures were spread on SD/-Leu/-Trp plates and the yeast colonies were then transferred onto SD/-Ade/-His/-Leu/-Trp/X-*α*-gal plates for assessing *β*-galactosidase activity. Positive and negative controls were performed in parallel.

### 2.4. *In Vitro* Binding Assay of PRAK and DJ-1

pGEX-KG-DJ-1 and pET-14b-PRAK were transformed into* E. coli* BL21 strain to produce GST-tagged DJ-1 fusion protein and His-tagged PRAK fusion protein, respectively. GST-DJ-1 was purified with GST-bind resin (Novagen) and eluted by reduced glutathione. His-PRAK was purified with Ni-NTA resin (Qiagen) and eluted by elution buffer (50 mM NaH_2_PO_4_, 300 mM NaCl, and 250 mM imidazole, pH 8.0). After incubation with either GST-DJ-1 fusion protein or GST, His-PRAK fusion protein was pulled down with Ni-NTA beads, and the precipitate was separated by SDS-PAGE.

### 2.5. *In Vitro* DJ-1 Phosphorylation Assay

GST-DJ-1, His-PRAK, and His-p38 fusion proteins were purified as described above. GST-DJ-1 was coincubated with either His-PRAK or His-p38 in the kinase assay buffer containing 25 mM Tris-HCl (pH 7.5), 5 mM *β*-glycerophosphate, 2 mM DTT, 0.1 mM Na_3_VO_4_, 10 mM MgCl_2_, and 2 *μ*M ATP (PhosphoDetect phosphoserine detection kit, Calbiochem) at 37°C for 1 hr. The samples were then separated by SDS-PAGE, transferred onto nitrocellulose membranes, and probed with anti-Ser phosphorylation antibody (Calbiochem).

### 2.6. Cell Cultures and Transfection

Human HEK293, NIH3T3, Hela, PRAK^+/+^, and PRAK^−/−^ MEF cells (kindly provided by Dr. Jiahuai Han, The Scripps Research Institute, La Jolla) were maintained at 37°C in DMEM supplemented with 10% FCS, penicillin (100 units/mL), streptomycin sulfate (100 *μ*g/mL), and glutamine (2 mM). All culture medium and reagents used for cell cultures were purchased from Invitrogen. For immunoprecipitation experiment, either HEK293 cells or NIH3T3 cells were cotransfected with pcDNA3-HA-PRAK and pcDNA3-Flag-DJ-1 plasmids for 24 hrs using Lipofectamine 2000 (Invitrogen) according to the manufacturer's instructions. For immunocytochemistry, NIH3T3 cells were cotransfected with either plasmids of pcDNA3-HA-PRAK and pEGFP-DJ-1 or plasmids of pCDNA3-HA-PRAK and pcDNA3-Flag-DJ-1 for 24 hrs using Lipofectamine 2000 (Invitrogen). For FRET assay, Hela cells were cotransfected with pECFP-DJ-1 and pEYFP-PRAK plasmids for 24 hrs using Lipofectamine 2000 (Invitrogen).

### 2.7. Immunoprecipitation and Immunoblotting

After transfection, human HEK293 cells were lysed in lysis buffer containing 150 mM NaCl, 1 mM EDTA, 1 mM EGTA, 1% Triton X-100, 2.5 mM sodium pyrophosphate, 1 mM *β*-glycerolphosphate, 1 mM Na_3_VO_4_, and a mixture of protease inhibitors and phosphatase inhibitors (Roche). Equal amounts of extracted protein were mixed with 10 *μ*L anti-Flag M2 beads (Sigma) and incubated on ice for 6 hrs. The samples were spun briefly and washed five times with lysis buffer containing 0.1% Tween-20. Loading buffer (20 *μ*L) was added to each sample and boiled for 5 min. The samples were then separated by SDS-PAGE, transferred onto nitrocellulose membranes, and probed with either anti-HA antibody (Cell Signaling Technology) or anti-Flag M2 antibody (Stratagene).

PRAK^+/+^ cells and PRAK^−/−^ cells were treated with H_2_O_2_ (300 *μ*M) for different time periods, and nonstimulated Hela cells were lysed as described above. Protein A/G beads (20 *μ*L) (sigma) were incubated with 2 *μ*g anti-DJ-1 antibody (Abcam), 2 *μ*g anti-PRAK antibody (BD Biosciences), or 2 *μ*g nonspecific IgG antibody (BD Biosciences) at 4°C for 6 hrs, spun briefly, and washed five times with lysis buffer containing 0.1% Tween-20. Equal amounts of extracted protein from PRAK^+/+^ cells, PRAK^−/−^ cells, and Hela cells were incubated with 20 *μ*L protein A/G beads coupled with anti-DJ-1 antibody, anti-PRAK antibody, or IgG on ice for 6 hrs. The samples were spun briefly and washed five times with lysis buffer containing 0.1% Tween-20. Loading buffer (20 *μ*L) was added to each sample and boiled for 5 min. The samples were then subjected to immunoblot analysis with anti-Ser phosphorylation antibody (Calbiochem), anti-DJ-1 antibody (Abcam), or anti-PRAK antibody (BD Biosciences).

NIH3T3 cells were lysed after transfection as described above. The resultant lysates were centrifuged and supernatants containing the cytoplasmic proteins were collected. For nuclear protein extraction, the pellets were further lysed in nuclear extraction buffer containing 20 mM Hepes, pH7.9, 420 mM NaCl, 1.5 mM MgCl_2_, 0.2 mM EDTA, 1 mM DTT, 25% glycerol, and a mixture of protease inhibitors and phosphatase inhibitors (Roche). Equal amounts of protein extracted from either cytoplasm or nucleus were subjected to immunoblot analysis.

### 2.8. Immunofluorescence Assay

NIH3T3 cells were cotransfected with pcDNA-HA-PRAK, pcDNA3-Flag-DJ-1, and PEGFP-DJ-1 for 24 hrs. PRAK^+/+^ cells and PRAK^−/−^ cells were starved for 48 hrs and were further challenged with H_2_O_2_ (300 *μ*M) for 6 hrs. Cells were fixed in 4% paraformaldehyde for 10 min, washed twice with PBS, and permeabilized with 0.1% sodium tetrahydroborate for 5 min. After being washed three times with PBST (PBS + 0.2%Triton X-100) and blocked with 3% BSA for 1 hr, cells were incubated with the indicated antibodies diluted in 3% BSA at room temperature for 1 hr and washed three times with PBST. Cells were further incubated with the indicated secondary antibodies diluted in 3% BSA at room temperature for 1 hr and washed three times with PBST. Cell nucleus was stained with 10 *μ*M DAPI. Fluorescent images were recorded and analyzed using a fluorescence microscope (DMRA2, Leica) equipped with FW4000 software.

### 2.9. FRET Analysis

Hela cells were cotransfected with pECFP-DJ-1 and pEYFP-PRAK using Lipofectamine 2000 (Invitrogen). The live cells were imaged using an inverted fluorescence microscope (Zeiss Axiovert 200 M) 36 hrs after transfection. The donor fluorophore (CFP) was excited at 436/25 nm and fluorescence emission was detected in a bandwidth of 480/40 nm (CFP channel), whereas the acceptor fluorophore (YFP) was excited at 500/25 nm and fluorescence emission was detected in a bandwidth of 535/30 nm (YFP channel). FRET excitation was conducted at 436/25 nm and fluorescence emission was detected in a bandwidth of 535/30 nm (FRET channel). To correct fluorescence bleed through into the FRET channel, cells transfected with either pECFP-DJ-1 alone or pEYFP-PRAK alone were used to determine the donor or the acceptor correction factor. Images of CFP-DJ-1 and YFP-PRAK expression in cotransfected cells were sequentially acquired with the donor (CFP) channel, acceptor (YFP) channel, and FRET channel under identical conditions. The image obtained with the FRET channel was evaluated using Carl Zeiss AxioVision FRET 4.6 software and values of FRET were calculated as described previously [28].

### 2.10. Cell Viability Assay

PRAK^+/+^ and PRAK^−/−^ cells were plated in 96-well tissue culture plates (2.5 × 10^4^ cells per well) and exposed to H_2_O_2_ (300 *μ*M) for different time periods. Cell viability was quantified using a Cell Titer 96 Aqueous One solution cell proliferation assay kit (Promega) with a HTS7000 Bio-Assay Reader (Perkin Elmer).

### 2.11. Statistical Analysis

All data are expressed as mean ± SD. Statistical analysis was performed by the Student's *t*-test and ANOVA. Differences were judged statistically significant when the *P* value was less than 0.05.

## 3. Results

### 3.1. Interaction of DJ-1 and PRAK in Yeast

**Figure 1:**
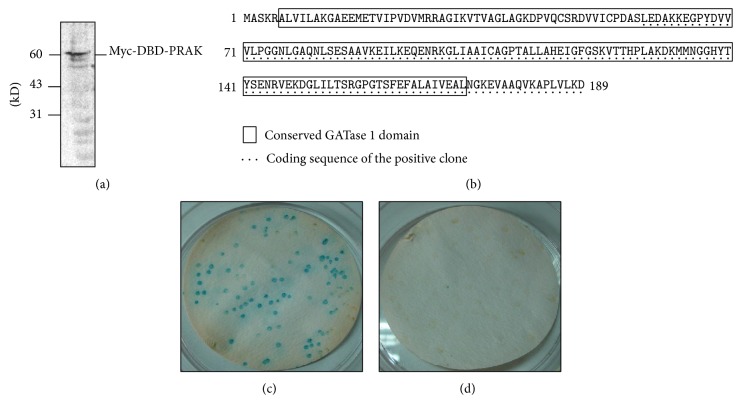
Interaction of DJ-1 and PRAK in yeast. (a) Myc-DBD-PRAK fusion protein detected by Western blot using an antibody against Myc. (b) The coding sequence of a positive clone was identical to the C-terminal (174~567 bp) of* Homo sapiens* DJ-1. The underdot-lined region represents the coding sequence (58–189 aa) of the positive clone that interacts with PRAK. (c) and (d) *β*-Galactosidase activity of yeast clones either expressing AD-DJ-1 and DBD-PRAK fusion proteins (c) or expressing AD-DJ-1 and DBD proteins (d) on SD/-Ade/-His/-Leu/-Trp/X-*α*-gal plates.

To screen the PRAK-binding proteins, we amplified a full-length human PRAK cDNA (1415 bp) from pcDNA3-HA-PRAK by PCR and subcloned it into the pGBKT7 vector. pGBKT7-PRAK was transformed into the yeast strain AH109 and placed on SD/-Trp plates, which expresses Myc-DBD-PRAK fusion protein as confirmed by Western blot analysis (Figure 1(a)).

A Gal4-based yeast two-hybrid system was used to identify proteins that interact with PRAK. The pGBKT7-PRAK plasmid was transformed into the yeast strain AH109 as the bait and a human heart cDNA library was previously transformed into the yeast strain Y187 as the prey. Approximately 1 × 10^6^ transformants were screened and a total of seven positive clones were obtained. NCBI blast results revealed that clone 4 was identical to the coding sequence (58–189 aa) of human DJ-1 (Figure 1(b)).

To further test the interaction between DJ-1 and PRAK, yeast strains AH109 expressing active domain- (AD-) fused DJ-1 and Y187 expressing DNA binding domain- (DBD-) fused PRAK were mixed, mated for 24 hrs, and plated on SD/-Leu/-Trp plates. After the yeast colonies were transferred onto SD/-Ade/-His/-Leu/-Trp/X-*α*-gal plates, *β*-galactosidase activity was measured to assess the interaction between DJ-1 and PRAK. Yeasts transformed with both DBD-PRAK and AD-DJ-1 expressed the LacZ phenotype (Figure 1(c)), whereas yeasts transformed with DBD and AD-DJ-1 failed to show any LacZ activity (Figure 1(d)), confirming that DJ-1 interacts with PRAK, but not DBD, in yeast.

### 3.2. *In Vitro* and* In Vivo* Interaction between DJ-1 and PRAK

**Figure 2:**
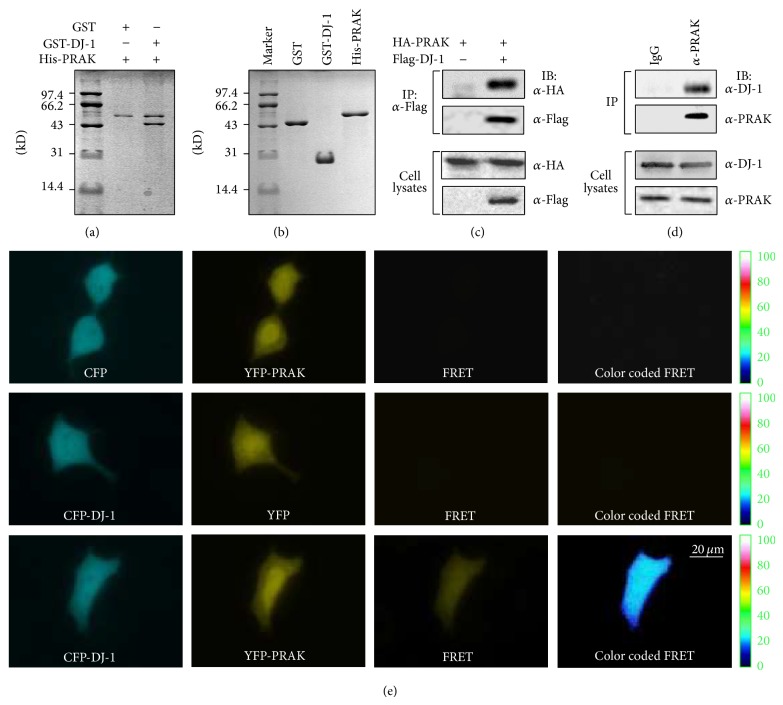
Interaction between PRAK and DJ-1* in vitro* and* in vivo*. (a) SDS-PAGE analysis of interaction of His-PRAK with either GST-DJ-1 or GST* in vitro*. The protein bands are visualized by Coomassie blue staining. (b) The equal input of His-PRAK, GST-DJ-1, and GST on SDS-PAGE. (c) HEK293 cells were cotransfected with pcDNA3/HA-PRAK and pcDNA3/Flag-DJ-1. pcDNA3-Flag was used as the control. Cell lysates were precipitated with anti-flag M2 beads, and both immunoprecipitates (upper) and cell lysates (lower) were immunoblotted with either anti-HA or anti-Flag antibodies. (d) Cell lysates from naive Hela cells were precipitated with protein A/G beads coupled with IgG (left) or anti-PRAK antibody (right). Both immunoprecipitates (upper) and cell lysates (lower) were immunoblotted with either anti-DJ-1 or anti-PRAK antibodies. (e) CFP-DJ-1 and YFP-PRAK were coexpressed in Hela cells followed by observation with different fluorescence channels CFP, YFP, and FRET. The FRET efficiency depicted as a color-coded scale ranging from 0 to 100%. Coexpression of either CFP and YFP-PRAK or YFP and CFP-DJ-1 in Hela cells was used as the control.

Glutathione S-transferase- (GST-) tagged DJ-1 and His-tagged PRAK fusion proteins were expressed in* E. coli*, respectively. Purified His-PRAK fusion protein was mixed with either GST-DJ-1 fusion protein or GST and further pulled down by nickel-nitrilotriacetic acid (Ni-NTA) precipitation and separated by SDS-PAGE. As shown in Figures 2(a) and 2(b), His-PRAK specifically bound to GST-DJ-1, but not GST,* in vitro*.

To assess the interaction of PRAK with DJ-1* in vivo*, human HEK293 cells were transfected with pcDNA3-HA-PRAK plasmid with or without pcDNA3-Flag-DJ-1 plasmid. Twenty-four hours after transfection, cell extracts were prepared and subjected to immunoprecipitation with an anti-Flag antibody. The precipitates were then blotted with an anti-HA antibody. HA-PRAK was coprecipitated with Flag-DJ-1 in cells cotransfected with Flag-DJ-1 and HA-PRAK, but not in cells transfected with HA-PRAK alone (Figure 2(c)), indicating that PRAK specifically binds to DJ-1 in HEK293 cells.

To examine whether PRAK binds to DJ-1 under physiological conditions, cell extracts prepared from Hela cells were immunoprecipitated with an anti-PRAK antibody or a nonspecific IgG, and the precipitates were further immunoblotted against an anti-DJ-1 antibody. The anti-PRAK antibody did precipitate PRAK, and furthermore, DJ-1 was detected in the precipitates with the anti-PRAK antibody but not with a control IgG (Figure 2(d)). These data clearly indicate that there is a constitutive binding of PRAK with DJ-1 in nonstimulated cells.

To further confirm the above finding in live cells, we constructed a pair of plasmids encoding either CFP-DJ-1 or YFP-PRAK to perform a fluorescence resonance energy transfer (FRET) assay. Hela cells were cotransfected with pECFP-DJ-1 and pEYFP-PRAK plasmids, and the coexpression of CFP and YFP was evaluated by FRET. As shown in Figure 2(e), cells cotransfected with both CFP-DJ-1 and YFP-PRAK depicted a distinct color-coded FRET region, with the efficiency ranging from 15% to 17%, which illustrates an interaction between PRAK and DJ-1. In contrast, cells cotransfected with either CFP and YFP-PRAK or YFP and CFP-DJ-1 failed to display any significant FRET (Figure 2(e)).

### 3.3. Colocalization between PRAK and DJ-1

**Figure 3:**
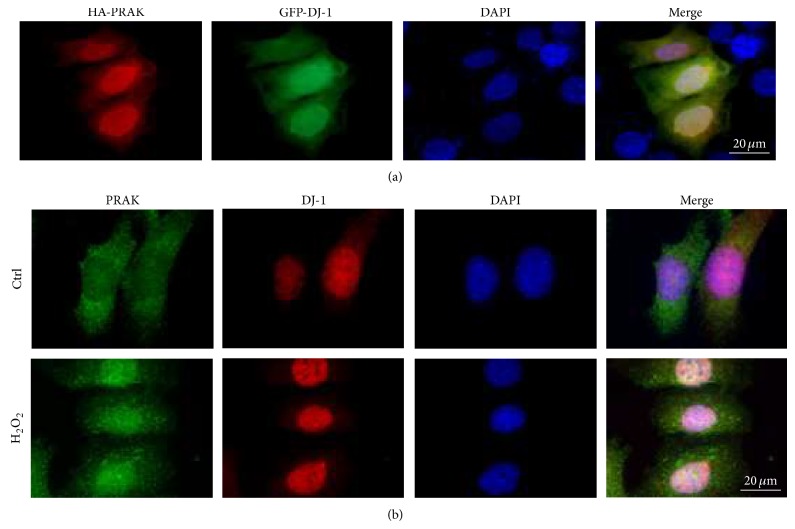
Intracellular colocalization between PRAK and DJ-1. (a) NIH3T3 cells were cotransfected with pcDNA3-HA-PRAK and pEGFP-DJ-1, stained with an anti-HA antibody and visualized with a Texas red-conjugated secondary antibody. (b) Naive PRAK^+/+^ cells (upper) or PRAK^+/+^ cells synchronized by serum starvation for 48 hrs and treated with 300 *μ*M H_2_O_2_ for 6 hrs (lower) were stained with the primary antibodies against PRAK and DJ-1 and visualized with FITC- and Texas red-conjugated secondary antibodies. Nuclei were stained with DAPI. Scale bar = 20 *μ*m.

To examine the intracellular localization of PRAK and DJ-1, NIH3T3 cells were cotransfected with pcDNA3-HA-PRAK and pEGFP-DJ-1 and further stained with an anti-HA antibody and visualized with a Texas red-conjugate secondary antibody. We observed that exogenously introduced HA-PRAK colocalized with GFP-DJ-1 in the nuclei of the NIH3T3 cells (Figure 3(a)). It has been reported that, in the normal circumstance, endogenous PRAK is mainly located in the cytoplasm of the cells [26], whereas the location of endogenous DJ-1 is cell cycle related and present in both cytoplasm and nucleus [27]. We stained PRAK^+/+^ cells with antibodies against PRAK and DJ-1 and FITC- and Texas red-conjugated secondary antibodies, to assess whether endogenous PRAK colocalized with endogenous DJ-1; however, there was no obvious colocalization between PRAK and DJ-1 observed in the nucleus (Figure 3(b)). In contrast, when PRAK^+/+^ cells were synchronized by serum starvation for 48 hrs and then treated with 300 *μ*M of H_2_O_2_ for 6 hrs, we did observe the colocalization of endogenous PRAK with DJ-1 in the nucleus (Figure 3(b)), indicating that, in response to oxidative stress, endogenous PRAK moves into the nucleus and colocalizes with DJ-1.

### 3.4. The Effect of PRAK on Subcellular Localization and Phosphorylation of DJ-1

**Figure 4:**
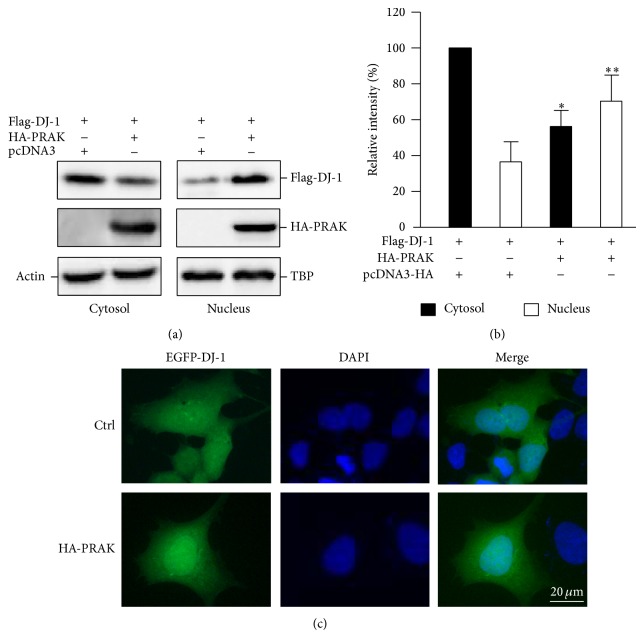
Overexpression of PRAK influences the intracellular distribution of DJ-1. (a) NIH3T3 cells were cotransfected with pcDNA3-Flag-DJ-1 and pcDNA3-HA-PRAK. pcDNA3-HA was used as the control. Both cytosolic and nuclear fractions of cell lysates were analyzed by Western blot with anti-Flag or anti-HA antibodies. TATA binding protein (TBP) and *β*-actin were used as internal controls for nuclear and cytosolic proteins, respectively. (b) The relative intensities of Flag-DJ-1 protein bands from Western blot were analyzed and data are expressed as the mean ± SD of four separate experiments. ^*^ *P* < 0.05 compared with Flag-DJ-1 in the cytosol fraction from cells transfected with pcDNA3-HA; ^**^ *P* < 0.05 compared with Flag-DJ-1 in the nuclear fraction from cells transfected with pcDNA3-HA. (c) NIH3T3 cells were cotransfected with pcDNA3-EGFP-DJ-1 and pcDNA3-HA-PRAK or pcDNA3-EGFP-DJ-1 and pcDNA3-HA as the control. Nuclei were stained with DAPI. Scale bar = 20 *μ*m.

NIH3T3 cells were transfected with pcDNA3-Flag-DJ-1, in combination with either pcDNA3-HA-PRAK or pcDNA-HA. Western blot analysis of cytoplasmic and nuclear extracts revealed that Flag-DJ-1 was mainly located in the cytoplasm when it was transfected alone; however, Flag-DJ-1 distributed in both cytoplasm and nucleus when it was cotransfected with HA-PRAK (Figures 4(a) and 4(b)), suggesting that overexpression of PRAK leads to a shift of DJ-1 from the cytoplasm to the nucleus. This finding was further supported by the results from fluorescent micrographs. GFP-DJ-1 was observed in the cytoplasm and nucleus when cells were transfected with pcDNA3-EGFP-DJ-1 alone (Figure 4(c)); however, more GFP-DJ-1 aggregated in the nucleus when cells were cotransfected with both pcDNA3-EGFP-DJ-1 and pcDNA3-HA-PRAK (Figure 4(c)).

**Figure 5:**
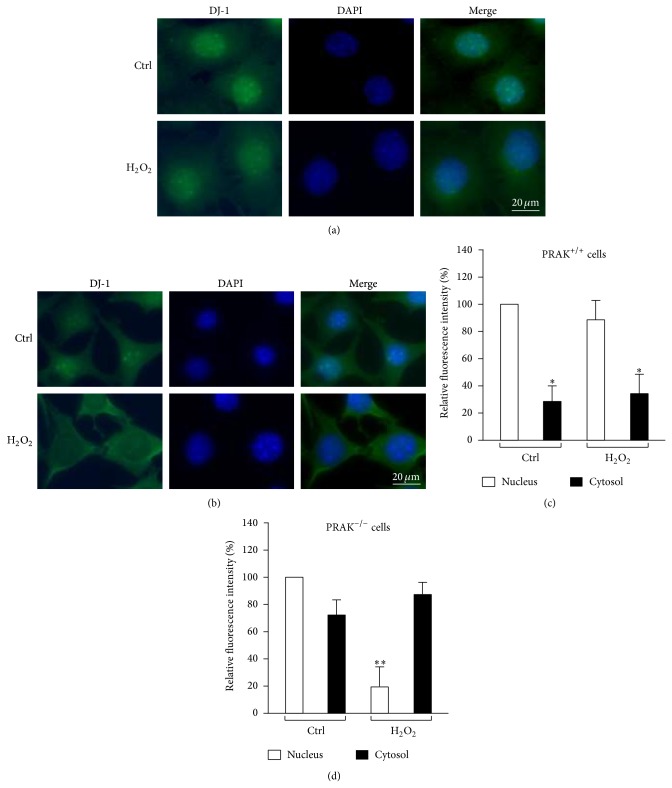
Effect of PRAK on DJ-1 nuclear localization under oxidative stress. (a) PRAK^+/+^ cells synchronized by serum starvation for 48 hrs and treated with culture medium (upper) or 300 *μ*M H_2_O_2_ (lower) for 6 hrs were stained with anti-DJ-1 antibody. (b) PRAK^−/−^ cells synchronized by serum starvation for 48 hrs and treated with culture medium (upper) or 300 *μ*M H_2_O_2_ (lower) for 6 hrs were stained with anti-DJ-1 antibody. Nuclei were stained with DAPI. Scale bar = 20 *μ*m. (c) and (d). The nuclear and cytoplasmic fluorescence intensities of DJ-1 in PRAK^+/+^ cells (c) and PRAK^−/−^ cells (d) were analyzed. Data are expressed as the mean ± SD of four separate experiments. ^*^ *P* < 0.05 compared with DJ-1 in the nucleus of control or H_2_O_2_-treated PRAK^+/+^ cells (c); ^**^ *P* < 0.05 compared with DJ-1 in the nucleus of control PRAK^−/−^ cells (d).

Next, we examined the localization of endogenous DJ-1 in PRAK^+/+^ and PRAK^−/−^ cells after the cells were synchronized by serum starvation for 48 hrs and treated with 300 *μ*M of H_2_O_2_ for 6 hrs. In PRAK^+/+^ cells, endogenous DJ-1 mainly located in the nucleus even after the cells were treated with H_2_O_2_ for 6 hrs (Figures 5(a) and 5(c)). However, in nonstimulated PRAK^−/−^ cells, more endogenous DJ-1 appeared in the cytoplasm when compared with nonstimulated PRAK^+/+^ cells (Figures 5(b) and 5(d)). Furthermore, most endogenous DJ-1 in PRAK^−/−^ cells translocated into the cytoplasm from the nucleus after the cells being treated with H_2_O_2_ for 6 hrs (Figures 5(b) and 5(d)).

**Figure 6:**
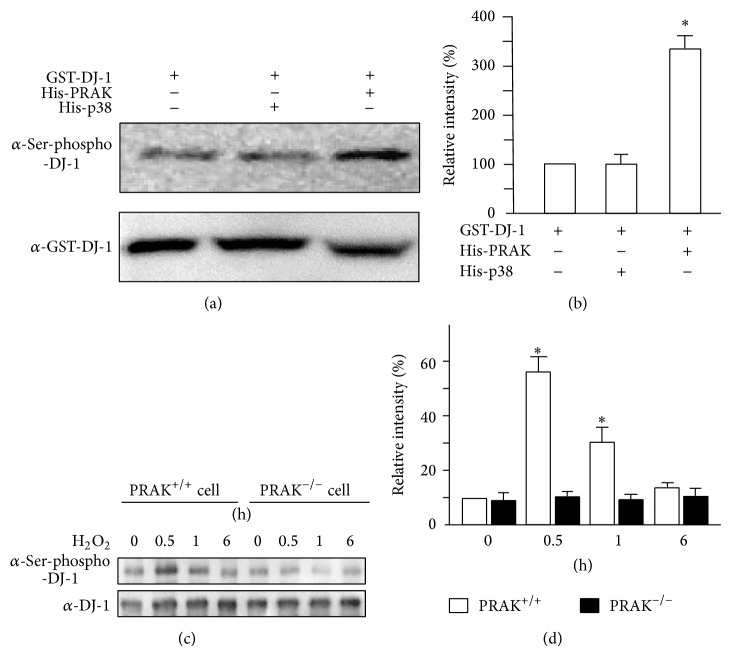
PRAK phosphorylates DJ-1 both* in vitro* and* in vivo*. (a) GST-tagged DJ-1 was coincubated with His-tagged PRAK or His-tagged p38 and further analyzed by Western blot. (b) The relative intensities of phosphorylated DJ-1 were analyzed and data are expressed as the mean ± SD of three separate experiments. ^*^ *P* < 0.05 compared with GST-DJ-1 coincubated with His-p38. (c) PRAK^+/+^ and PRAK^−/−^ cells were treated with 300 *μ*M H_2_O_2_ for different time periods. The expression of phosphorylated and total DJ-1 was assessed by Western blot analysis. (d) The relative intensities of phosphorylated DJ-1 were analyzed and data are expressed as the mean ± SD of three separate experiments. ^**^ *P* < 0.05 compared with PRAK^−/−^ cells.

To assess whether PRAK can directly phosphorylate DJ-1, GST-tagged DJ-1 was incubated with His-tagged PRAK or p38 fusion proteins. Coincubation of His-PRAK but not His-p38 with GST-DJ-1 induced phosphorylation of DJ-1 (Figures 6(a) and 6(b)). To further validate our* in vitro* finding, PRAK^+/+^ and PRAK^−/−^ cells were treated with 300 *μ*M H_2_O_2_ for different time periods. In contrast to PRAK^−/−^ cells, H_2_O_2_-challenged PRAK^+/+^ cells displayed a substantially increased expression of phosphorylated DJ-1 (Figures 6(c) and 6(d)), indicating that PRAK phosphorylates DJ-1 in response to H_2_O_2_-induced oxidative stress.

### 3.5. PRAK Facilitates DJ-1 to Sequester Daxx in the Nucleus and Prevent Cell Death

**Figure 7:**
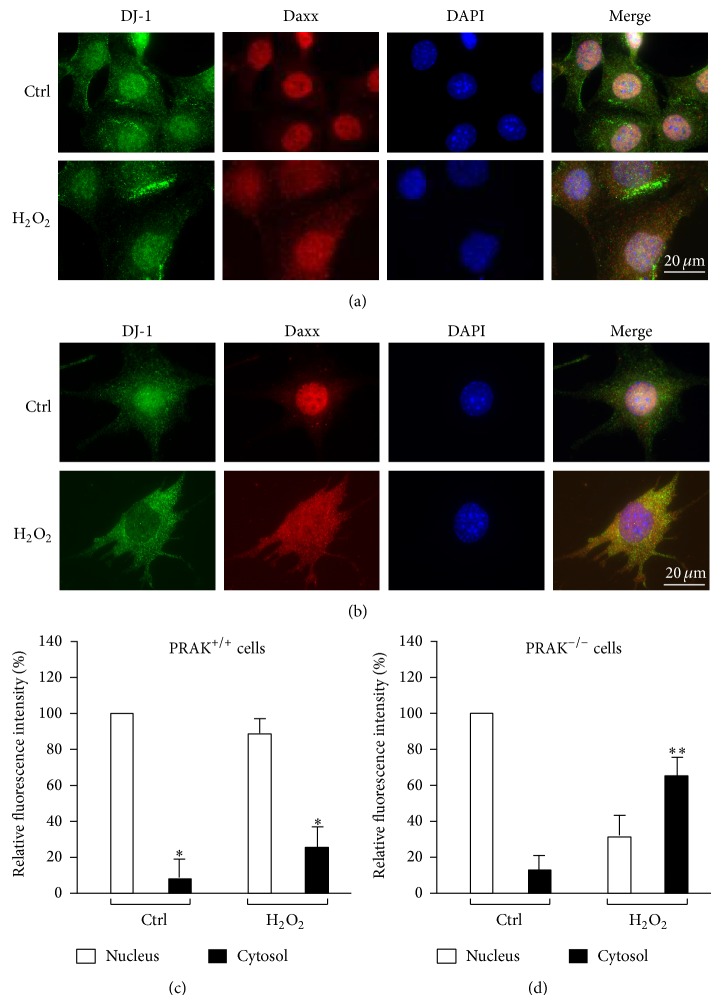
PRAK helps DJ-1 to sequester Daxx in the nucleus. (a) PRAK^+/+^ cells synchronized by serum starvation for 48 hrs and treated with culture medium (upper) or 300 *μ*M H_2_O_2_ (lower) for 6 hrs were stained with antibodies against DJ-1 and Daxx and further visualized with FITC- and Texas red-conjugated secondary antibodies. (b) PRAK^−/−^ cells synchronized by serum starvation for 48 hrs and treated with culture medium (upper) or 300 *μ*M H_2_O_2_ (lower) for 6 hrs were stained with antibodies against DJ-1 and Daxx and further visualized with FITC- and Texas red-conjugated secondary antibodies. Nuclei were stained with DAPI. (c) and (d). The nuclear and cytoplasmic fluorescence intensities of Daxx in PRAK^+/+^ cells (c) and PRAK^−/−^ cells (d) were analyzed. Data are expressed as the mean ± SD of four separate experiments. ^*^ *P* < 0.05 compared with Daxx in the nucleus of naive or H_2_O_2_-treated PRAK^+/+^ cells (c); ^**^ *P* < 0.05 compared with Daxx in the cytoplasm of naive PRAK^−/−^ cells (d).

Previous studies reported that Daxx interacts with apoptosis signal-regulating kinase 1 (ASK1) and causes activation of this kinase, which subsequently triggers cell death [29], whereas DJ-1 can hamper the interaction between Daxx and ASK1 by recruiting Daxx in the nucleus, thereby inhibiting ASK1 activation and cell death [30]. We found that endogenous DJ-1 normally located in the nuclei of PRAK^+/+^ cells; however, in PRAK^−/−^ cells, DJ-1 translocated from the nucleus into the cytoplasm following H_2_O_2_ treatment (Figure 5). Based on these findings, we hypothesized that, under oxidative stress, DJ-1, in the absence of PRAK, is unable to sequester Daxx in the nucleus, and more Daxx translocate into the cytoplasm, thereby causing ASK1 activation and cell death. To confirm this, we assessed DJ-1 and Daxx localization in both PRAK^+/+^ and PRAK^−/−^ cells following H_2_O_2_ treatment. In PRAK^+/+^ cells, DJ-1 and Daxx colocalized in the nucleus (Figures 7(a) and 7(c)). After the cells were treated with 300 *μ*M H_2_O_2_ for 6 hrs, DJ-1 still remained in the nucleus and the majority of Daxx was kept in the nucleus despite a small amount of Daxx which translocated into the cytoplasm (Figures 7(a) and 7(c)). In contrast, most DJ-1 in PRAK^−/−^ cells translocated into the cytoplasm in response to the H_2_O_2_ challenge and failed to sequester Daxx in the nucleus (Figures 7(b) and 7(d)). As a result, Daxx translocated from the nucleus into the cytoplasm (Figures 7(b) and 7(d)).

**Figure 8:**
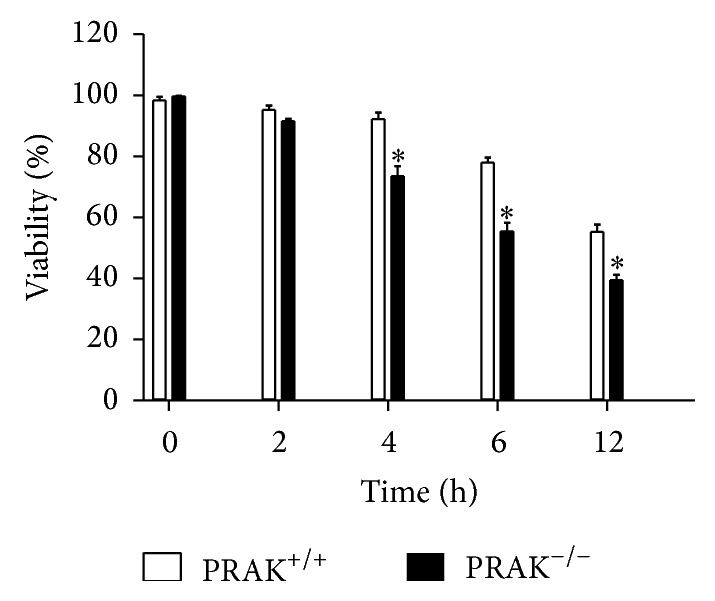
Cell viability in PRAK^+/+^ and PRAK^−/−^ cells challenged with H_2_O_2_-induced oxidative stress. PRAK^+/+^ and PRAK^−/−^ cells were treated with 300 *μ*M H_2_O_2_ for different time periods. Cell viability was assessed as described in Section 2. Data are expressed as the mean ± SD of triplicate samples and representative of at least four to six independent experiments. ^*^ *P* < 0.05 compared with PRAK^+/+^ cells.

To further examine the influence of cytoplasmic translocation of Daxx, observed in H_2_O_2_-treated PRAK^−/−^ cells, on cell survival, we incubated both PRAK^+/+^ and PRAK^−/−^ cells with 300 *μ*M H_2_O_2_ for different time periods. As shown in Figure 8, PRAK^−/−^ cells exhibited significantly impaired ability to survive from H_2_O_2_-induced oxidative stress when compared to PRAK^+/+^ cells.

## 4. Discussion

DJ-1, first identified by Nagakubo et al. [27] as a mitogen-dependent oncogene product, is ubiquitously expressed in almost all human tissues as homodimers and participates in many physiological and pathological processes including tumorigenesis [31–33], fertilization [34, 35], regulation of the androgen receptor [36–40], posttranslational modification of protein SUMO-1, a ubiquitin-like modifier [41], oxidative stress [42–44], and the development of Parkinson's disease [45–49]. However, it is undefined whether DJ-1 is a downstream interacting target for PRAK. In the present study, using a yeast two-hybrid system, we identified that DJ-1 is a potential PRAK interacting partner. A pull-down assay demonstrated that His-PRAK exclusively bound to GST-DI-1. Immunoprecipitation and immunoblotting data from human HEK293 cells revealed that PRAK was coprecipitated with DJ-1 in cells cotransfected with pCDNA3-HA-PRAK and pCDNA3-Flag-DJ-1 plasmids but not in cells transfected with pCDNA3-HA-PRAK alone. In addition, a constitutive binding of endogenous PRAK with DJ-1 was observed in nonstimulated Hela cells as confirmed by immunoprecipitation with anti-PRAK antibody and immunoblotting with anti-DJ-1 antibody. Using a FRET-based technique, we further illustrated an interaction between PRAK and DJ-1 in Hela cells. These results clearly demonstrate that PRAK binds to and interacts with DJ-1 both* in vitro* and* in vivo*.

It has been shown that endogenous PRAK is mainly located in the cytoplasm, whereas exogenous PRAK predominates in the nucleus [26]. On the other hand, endogenous DJ-1 is present in both cytoplasm and nucleus [27]. However, it is unclear whether PRAK preferentially colocalizes with DJ-1, thus affecting the intracellular distribution of DJ-1. We first examined the intracellular colocalization of either exogenously introduced or endogenous PRAK and DJ-1. We cotransfected NIH3T3 cells with pCDNA3-HA-PRAK and pEGFP-DJ-1 plasmids and observed colocalization of exogenously introduced PRAK with DJ-1 in the nucleus. Although there was no apparent colocalization of endogenous PRAK with DJ-1 found in nonstimulated cells, we did observe that endogenous PRAK in PRAK^+/+^ cells colocalized with DJ-1 in the nucleus in response to H_2_O_2_-induced oxidative stress. To further examine the influence of PRAK on subcellular localization of DJ-1, we transfected NIH3T3 cells with pcDNA3-Flag-DJ-1 in the presence or absence of pcDNA3-HA-PRAK. When cells were transfected with Flag-DJ-1 alone, the exogenously introduced DJ-1 was mainly located in the cytoplasm. However, when cells were cotransfected with both Flag-DJ-1 and HA-PRAK, more exogenously introduced DJ-1 translocated from the cytoplasm into the nucleus. Similarly, endogenous DJ-1 in PRAK^+/+^ cells was mainly located in the nucleus even after the cells were treated with H_2_O_2_ for 6 hrs; in contrast, most endogenous DJ-1 in PRAK^−/−^ cells translocated from the nucleus into the cytoplasm in response to H_2_O_2_ challenge. These results demonstrate that PRAK preferentially colocalizes with DJ-1 and helps DJ-1 to localize in the nucleus in response to oxidative stress. On the other hand, it has been reported that DJ-1 can shuttle between cytoplasm and nucleus [27], but it contains no NLS [50], indicating that there must be some other protein(s) which interact with DJ-1 and decide the subcellular localization of DJ-1. Our data support the notion that PRAK is one of such candidates that interacts with DJ-1 and assists its shuttling between nucleus and cytoplasm. It is important to clarify whether interaction of PRAK with DJ-1, in addition to facilitating the intracellular localization of DJ-1, also leads to DJ-1 phosphorylation. Using an* in vitro* assay system, we found that phosphorylation of DJ-1 was achieved only when GST-DJ-1 was coincubated with His-PRAK fusion protein. Furthermore, a substantially increased phosphorylation of endogenous DJ-1 in response to H_2_O_2_-induced oxidative stress was observed in PRAK^+/+^ cells but not in PRAK^−/−^ cells. These data clearly demonstrate a PRAK-dependent phosphorylation of DJ-1.

Next, we attempted to clarify the biological significance of sequestering DJ-1 in the nucleus by PRAK in response to oxidative stress. Recent studies have revealed that DJ-1 functions as a new type of H_2_O_2_ scavenger [51]; however, DJ-1 protects against oxidative stress-induced cell death via its sequestration of Daxx, a death protein in the nucleus, thus preventing subsequent activation of ASK1-mediated cell death pathway, rather than its direct effect of scavenging H_2_O_2_ [30]. Based on these findings, we hypothesized that PRAK facilitates DJ-1 to sequester Daxx in the nucleus, thus protecting against oxidative stress-induced cell death. To test this, we treated cells with H_2_O_2_ and observed that, in PRAK^+/+^ cells, the majority of DJ-1 and Daxx were still colocalized in the nucleus, whereas most DJ-1 and Daxx in PRAK^−/−^ cells translocated from the nucleus into the cytoplasm, demonstrating that, without PRAK, DJ-1 fails to sequester Daxx in the nucleus in response to oxidative stress; as a result, more Daxx translocate into the cytoplasm where it triggers ASK1-associated cell death pathway. Consistent with this, we observed a substantially increased cell death in H_2_O_2_-treated PRAK^−/−^ cells compared to H_2_O_2_-treated PRAK^+/+^ cells. In supporting our finding, a recent study by Han and colleagues [52] has reported that PRAK plays a key role in* ras*-induced senescence and tumor suppression by directly phosphorylating and activating the tumor-suppressor protein p53, indicating that PRAK possesses a diverse range of biological functions dependent on its downstream interacting partners.

Taken together, we identified DJ-1 as a novel interacting protein for PRAK. PRAK preferentially colocalizes with DJ-1 and leads to DJ-1 activation, which in turn facilitates DJ-1 to sequester Daxx in the nucleus, preventing oxidative stress-induced cell death. Further elucidation of molecular mechanisms underlying the interaction of PRAK, DJ-1, and Daxx may unravel a novel cytoprotective function of PRAK in response to oxidative stress.

## Abbreviations

AD: Activation domain
ASK1: Apoptosis signal-regulating kinase 1
DBD: DNA binding domain
ERK3: Extracellular signal-regulated kinase 3
FRET: Fluorescence resonance energy transfer
GST: Glutathione S-transferase
HSP27: Heat shock protein 27
MAPK: Mitogen-activated protein kinases
MAPKAPK5: Mitogen-activated protein kinase activated protein kinase 5
NES: Nuclear export sequence
Ni-NTA: Nickel-nitrilotriacetic acid
NLS: Nuclear localization sequence
PRAK: p38 regulated/activated kinase.
